# Mediators of Inflammation in Topical Therapy of Skin Cancers

**DOI:** 10.1155/2019/8369690

**Published:** 2019-01-10

**Authors:** Vlad Mihai Voiculescu, Cristina Victoria Lisievici, Mihai Lupu, Cristina Vajaitu, Carmen Cristina Draghici, Alexandra Victoria Popa, Iulia Solomon, Teona Ioana Sebe, Maria Magdalena Constantin, Constantin Caruntu

**Affiliations:** ^1^Department of Dermatology, “ELIAS” University Emergency Hospital, Bucharest, Romania; ^2^Carol Davila University of Medicine and Pharmacy Bucharest, Romania, Bucharest, Romania; ^3^Dermatology Clinic, MedAs Medical Center, Bucharest, Romania; ^4^The Clinic of Plastic Surgery Reconstructive Microsurgery, Emergency Hospital Bucharest, Romania; ^5^2nd Department of Dermatology, “Colentina” Clinical Hospital, Bucharest, Romania; ^6^Department of Dermatology, Prof. “N Paulescu” National Institute of Diabetes, Nutrition and Metabolic Diseases, Bucharest, Romania

## Abstract

Taking into consideration that the immune system plays a very important role in the development of melanoma and non-melanoma skin cancers, which have a high prevalence in immunosuppressed patients and after prolonged ultraviolet radiation, the interest in developing novel therapies, in particular targeting the inflammation in cancer, has increased in the past years. The latest data suggest that therapies such as imiquimod (IMQ), ingenol mebutate (IM), 5-fluorouracil (5-FU), retinoids, and nonsteroidal anti-inflammatory drugs (NSAIDs) have been used with success in the topical treatment of some cancers. Herein, we review the topical treatment targeting the inflammation in skin cancer and the mechanisms involved in these processes. Currently, various associations have shown a superior success rate than monotherapy, such as systemic acitretin and topical IMQ, topical 5-FU with tretinoin cream, or IMQ with checkpoint inhibitor cytotoxic T lymphocyte antigen 4. Novel therapies targeting Toll-like receptor-7 (TLR-7) with higher selectivity than IMQ are also of great interest.

## 1. Introduction

Melanoma and non-melanoma skin cancers (NMSCs) have known an increase in incidence throughout the years as scientists estimate that over 1.3 million new cases/year of NMSC will be identified in the US, ultraviolet (UV) radiation being the most important risk factor for this type of cancer [1]. Risk factors for developing skin cancers, beside chronic UV exposure, include human papillomavirus (HPV) infection, immunosuppression, family history of skin cancer, and light skin [2, 3]. The most common forms of NMSC are basal cell carcinoma (BCC) and squamous cell carcinoma (SCC), representing 80% and 20%, respectively, of NMSC [4].

BCC's incidence is increasing by 10% every year among white people living in geographical areas with high sun exposure, like Australia [5–7]. Unlike SCC, which may be lethal, BCC is only aggressive through its local extension and has high recurrence rate if the surgical treatment is not properly carried out [8]. Although surgical treatment is the gold standard therapy for BCC, being chosen in 95% of the cases, a large range of other options has developed including topical administration of IMQ, 5-FU, IM, or photodynamic therapy [9–11].

While BCCs rarely metastasize (<1% of cases), this risk in SCCs is much higher (2-5% of cases), however still remaining remarkably lower than other types of cancer [12–16]. The earliest stage in which a SCC can be diagnosed is actinic keratosis (AK), known to invade only the epidermis of chronically sun-exposed skin areas and having a potential of <1% to 16% per year of progression to SCC [17–19]. The American Academy of Dermatology estimates that 60% of patients of at least 40 years old, who present a predisposition, develop at least one AK [20]. Risk factors for developing an AK are immunosuppression, ageing, and fair skin [21, 22]. AK treatment includes surgical (excision, dermabrasion, laser therapy, electrosurgery, and curettage) and nonsurgical treatment (5-FU, trichloroacetic acid, tretinoin, IM, and diclofenac) [1, 23, 24].

Melanoma, the most deadly form of skin cancer contributing to 10,000 deaths per year in the United States [25], is a type of tumor strongly related to inflammatory processes, due to the high levels of secreted cytokines and the production of ROS (reactive oxygen species) and RNS (reactive nitrogen species). Recent data suggests that the secreted cytokines have a paracrine role in the tumoral microenvironment and also promote tumoral growth. The expression of IL-1 stimulates angiogenesis and promotes tumoral growth [26]. During melanoma evolution, activated macrophages produce TGF-beta (transforming growth factor-beta), TNF-alpha (tumoral necrosis factor-alpha), IL-1 alpha (interleukin-1 alpha), arachidonate metabolites, and extracellular proteases, while melanocytes express IL-8 and VEGF-alpha (vascular endothelial growth factor-alpha), inducing angiogenesis [27].

It has been shown that the immune system plays a very important role in the development of NMSC, considering the fact that it has a high prevalence in immunosuppressed patients and after prolonged UV (ultraviolet) radiation (which induces skin immunosuppression) [1, 22, 28]. UV radiation induces skin immunosuppression through various mechanisms such as the following: it stimulates natural killer (NK) cells which are implicated in the mediation of antigen-specific immune suppression, it reduces the number and functionality of Langerhans cells, and it stimulates the production of various immunosuppressive cytokines and affects genes which regulate proteins like p53 that influence the cell cycle [29–32]. UVB induces mutations of the p53 tumor suppressor gene resulting in the accumulation of keratinocytes with a mutated p53 gene, which may progress to actinic keratosis (AK) and NMSC [33–37]. Therefore, stimulating the immune system might be an efficient therapeutic strategy, with intralesional interferon already being successfully used to treat AKs, BCCs, and small SCCs [5, 38–40].

Current literature confirms the idea that cancer may develop under specific environments generated by chronic inflammation. These cells suffer intrinsic genetic modifications, and the surrounding inflammatory status influences the neoplastic growth and spread. This condition favors the development of an immunosuppressive environment by recruiting suppressor cells, like CD4^+^, CD25^+^, FOXp3^+^ Treg (regulatory T cells), myeloid-derived suppressor cells, tumor-associated macrophages, and regulatory dendritic cells. Moreover, the neoplastic cells may escape the immune surveillance due to some mediators like TGF-beta and IL-10 [41].

TLRs are considered novel therapeutic drug targets, especially due to their potential role in the recognition of pathogen-associated molecular patterns (PAMPs) of different origins and generation of proinflammatory response during some inflammatory conditions. Even ssRNA-based medications targeting TLR-7 and TLR-8 have potent antitumor actions and reverse the immunosuppressive action of Tregs *via* dendritic cells and *via* inducing a Th1 immune response [42]. TLR signaling acts in two different directions regarding cancer therapy, because it appears that the tumor cells use the TLR's role in the tissue homeostasis to create proper conditions for growth and survival [43].

### 1.1. Imiquimod

Imiquimod (1-isobutyl-1*H*-imidazo[4,5-*c*]quinolin-4-amine)(IMQ) is a low-molecular-weight, novel synthetic compound and member of the imidazoquinoline family that binds to TLR-7 and -8, determining high levels of interferon-alpha (IFN-*α*), tumor necrosis factor alpha (TNF-*α*), and other interleukins (IL-6, IL-8, etc.) [44–46]. Its mechanisms of action are not fully understood, but some theories may explain a part of them. It has been suggested that IMQ activates Langerhans cell migration and determines contact hypersensitivity by stimulating cytokine expression and, as a result, enhances antigen presentation [46]. IMQ is an immune response modifier, offering topical, noninvasive, and nonsurgical therapeutic options for some dermatological diseases. IMQ is also able to induce tumor cell apoptosis, which may suggest that it can be used in patients with skin tumors, especially in those with small tumors, with low-risk locations, that are not eligible for other therapies [47].

The innate immune system comprises immune cells like monocytes, macrophages, neutrophils, dendritic cells, natural killer cells, mast cells, eosinophils, and basophils and also newly identified innate lymphoid cells and mucosal associated invariant T, *γδ*T cells, NKT cells, etc., and its humoral components, meaning the circulating complement system proteins/components, cytokines, and chemokines secreted by innate immune cells along with various antimicrobial peptides [48].

The innate immune cells express a large variety of pattern recognition receptors (PRRs) including TLRs which recognize the pathogen-associated molecular patterns (PAMPs). TLRs also have an impact on the adaptive immune response mediated by different types of T cells and B cells. It has been demonstrated that TLRs play an important role in sterile inflammatory diseases, from cancer to autoimmunity (systemic lupus erythematosus, rheumatoid arthritis, spondyloarthritis, multiple sclerosis, and myositis). They are also involved in the pathogenesis of multiple human cancers such as B cell malignancies, colorectal cancer, BCC, and bladder cancer [48, 49].

**Figure 1:**
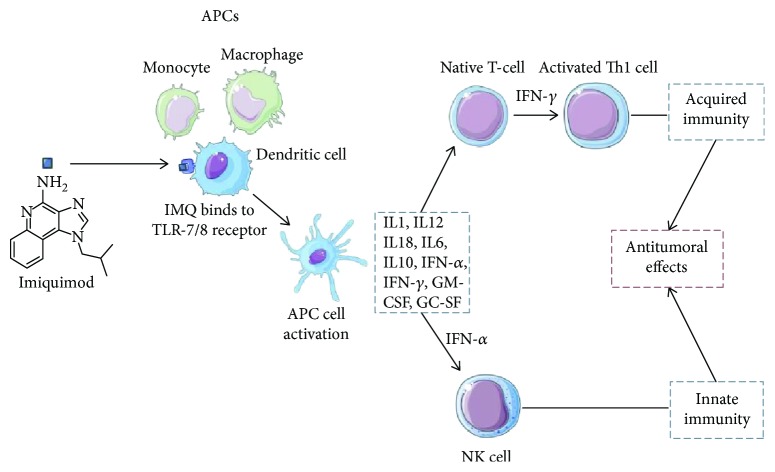
IMQ's primary mechanisms of action. APC: antigen-presenting cell; GC-SF: granulocyte colony-stimulating factor; GM-CSF: granulocyte-macrophage colony-stimulating factor; IFN: interferon; IMQ: imiquimod; IL: interleukin; TRL: Toll-like receptor; TNF: tumor necrosis factor.

It has been shown that TLR-7 can bind IMQ and virus-derived ssARN and is found in the structure of the endosomal membrane of dendritic cells, macrophages, monocytes, and mast cells [50–53]. By activating the TLRs, antigen-presenting cells (APCs) (monocytes, macrophages, B cells, and dendritic cells) are stimulated and a signaling cascade that recruits protein kinases and transcription factors is started. The result is the maturation and secretion of IL-1, IL-12, IL-18, IL-6, IL-10, and IFN-*α* by the target cells. These cytokines also stimulate the secretion of IFN-*γ* by native T cells, which leads to a Th1 lymphocyte-mediated immune response and the inhibition of Th2 cells (Figure 1) [46, 54–58].

IMQ also has the ability to induce 2′5′-oligoadenylate synthetase, leading to an activation of NK cells and perforin in cytotoxic T cells. The apoptotic effect is achieved through the activation of Bcl-2 (B cell lymphoma-2) proteins of the mitochondrial pathway [59].

Recent studies suggest that IMQ is also very useful in diseases associated with pathological neovascularization such as dysplastic nevi, melanoma, NMSCs, Kaposi's sarcoma, hemangioma of infancy, pyogenic granuloma, and angiosarcoma, as an inhibitor of angiogenesis. Its antiangiogenic activity is based on the ability to increase the secretion of IL-10 and IL-12 but also IFN's ability to decrease cellular production of some proangiogenic factors like b-FGF, IL-8, and urokinase plasminogen activator, to inhibit vascular motility and invasion, and to induce endothelial cell apoptosis [59]. Moreover, IP-10, the interferon-inducible protein 10, has an angiostatic effect [59, 60]. IL-12 inhibits endothelial proliferation and tube formation *in vitro* and angiogenesis *in vivo*, by upregulating IFN-*γ*, decreasing the production of VEGF and b-FGF (fibroblast growth factor), and inhibiting endothelial migration and invasion [59, 61]. The antiangiogenic mechanism of IL-10 is yet unknown, but the most probable theory is that it increases the expression of thrombospondin 1 and 2 inhibitors [49, 59].

Matrix metalloproteinases (MMP) are implicated in tumor growth, vessel formation, and metastasis [62–65]. Their role in vascular invasion and metastasis is based on their ability to cleave type IV collagen that can be found in the basement membrane [12]. This kind of activity can be stopped through MMP tissue inhibitors (TIMP), which are molecules that can bind to MMPs and inhibit their proteolytic activity, with TIMP-1 and TIMP-2 being the most important [66, 67]. It has been suggested that topical IMQ stimulates a 14-fold increase in TIMP-1 expression and a 5-fold reduction in MMP-8 [58, 59].

The advantages of the use of topical IMQ are that it is self-applied, it is a nonscarring procedure, and it is less expensive and less painful. Moreover, it can be used as an alternative on sensitive areas or lesions that involve large areas which are not susceptible to surgery [59].

Resiquimod, an imidazoquinoline, has been recently investigated as a topical adjuvant for skin cancer treatment. Although it has shown important positive results after topical treatment, the TLR-7 agonists may induce cardiac toxicity, when used at therapeutic regimens [47].

#### 1.1.1. Imiquimod and BCC

BCC is caused by aberrant activation of the hedgehog/glioma-associated oncogene pathway, mostly due to genetic inactivation of the protein patched homolog (PTCH) gene or activation of “smoothened.” Recent studies have shown that IMQ mechanisms of action include the stimulation of adenosine receptor/protein kinase A-mediated GLI phosphorylation, resulting in the inhibition of hedgehog signaling [68].

BCCs often express HLA class I molecules which will be recognized by reactive CD8 lymphocytes, but also monocytes, macrophages, and dendritic cells. The release of immunosuppressive cytokines, for example IL-10, may have an important role by impairing tumor cell recognition [47, 69].

A recent study has demonstrated that regression of BCC is associated with the activity of the innate immune response, with its origin in the macrophage-monocyte cells. Moreover, this response was associated with stimulation of apoptosis. As a result, more than 1300 genes which were differentially expressed after IMQ treatment were identified, most of them being involved in the immune response, and also a strong upregulation of genes involved in the apoptotic signaling pathway [47, 69]. An important aspect is the decrease in Bcl-2 expression, which means that cells become susceptible to apoptosis after IMQ treatment. First, IMQ stimulates the plasmacytoid dendritic cells in the epidermis and dermis in order to release IFN-alpha and other cytokines, resulting in activation of the innate immune system cells and release of oxygen reactive intermediates and other toxic molecules, all of this leading to the apoptosis of tumoral cells. They also suggested that this mechanism is related to destruction of the overlying epithelial cells resulting in typical erosions observed during IMQ treatment. An important observation is that T cell activation occurred later during treatment, suggesting that this is not the main factor during tumoral cell elimination [47, 69].

Berman et al. observed that IMQ-induced FasR- (Fas receptor-) mediated apoptosis may contribute to the effectiveness of IMQ 5% cream in the treatment of BCC. The expression of FasR leads to apoptosis via CD95 receptor-CD95 ligand (FasL) interaction, after which a cascade of events follows, including caspase activation. On the other hand, the BCC cells normally fail to express the Fas receptor, which may be responsible for their prolonged life, escaping apoptosis. Moreover, BCC cells strongly express FasL, which is associated with apoptosis of peritumoral T lymphocytes [69, 70]. After IMQ is applied topically to the skin, it modifies the immune response by inducing IFN-*α*, which, in the end, upregulates the expression of FasR and at the same time continues to express FasL, making the FasR-FasL-mediated apoptosis possible. In Berman et al.'s study, they examined the expression of FasR on BCC after short-term exposure to IMQ 5% cream or vehicle, applied five times per week for approximately 2 weeks. Histology showed that BCC cells were present in all of the vehicle-treated BCCs and in 4/5 of the IMQ-treated BCCs. The FasR was expressed in three quarters of the IMQ-treated BCCs and in none of the vehicle-treated tumors. None of the vehicle-treated BCCs presented T-lymphocytes near the BCCs cells, compared to all three IMQ-treated BCCs which expressed FasR [70].

The treatment of superficial BCC implies a regimen of 5 applications/week for 6 weeks (5% IMQ cream). This application rate has proven to histologically eradicate a superficial BCC up to 82% at a 3-month follow-up and 89% at a 39-month follow-up [71–73]. A 5-year follow-up from the SINS study revealed that there were no recurrences, years after topical treatment with IMQ, in BCC lesions. One major limitation of this study is the fact that follow-up at 3-5 years was most likely made in the community by the general practitioner, who might not be as vigilant in identifying subtle changes. Regardless, this study has relevant results, considering the fact that most treatment failures are identified early, local adverse effects were not severe enough to determine withdrawal from the study and also treatment response seems to be long-lasting. In those cases in which recurrence did occur, treatment of the lesion was not influenced by the first therapeutical option [74]. An exhaustive review of the literature confirmed that cryotherapy, photodynamic therapy (PDT), topical IMQ, and 5-FU are valid alternatives for low-risk superficial BCCs [10]. Other studies show that topical IMQ 5% therapy has superior success rates than 5-FU and PDT [75, 76] even though there seems to be no link between tumor thickness and success rate regarding the three options mentioned above [77].

Studies show that IMQ is more efficient in BCCs localized on the face compared to the ones on the trunk, which is reassuring considering the high recurrence rate of facial BCC [71]. Vun et al. found no correlation between the severity of the reactions at the application site (itching, crusting) and the response rate [71]. On the other hand, Chakrabarty and Geisse observed a positive association between the dosing frequency and the response rate,and also the occurrence of local side effects. Moreover, this study showed that the occlusion of the skin after IMQ application does not enhance the efficacy, but instead it may produce severe side effects [46].

This kind of topical treatment should be seriously taken into consideration when facing a lesion with both health and aesthetic concerns. Although there are some side effects of IMQ topical therapy, they are usually mild and well tolerated [71].

Bostanci et al. have proposed the use of IMQ not only for superficial BCC, for which it is approved, but also for other histological subtypes, with good long-term cosmetic results. The authors included tumors greater than 1 cm in diameter with various subtypes, including aggressive variants (infiltrative, metatypical, and solid). A recent trial which compared the surgical results versus IMQ 5% cream in patients with nodular and superficial BCC concluded that although surgery was superior, IMQ also showed promising results. The cosmetic appearance after 3 years was superior in the IMQ group vs surgical group (60.6% vs 35.6%). The histologic clearance rate was more than 80% among nodular BCCs larger than 1 cm in diameter. However, for nasal localization of the BCC, the results were not as satisfactory, with a long-term response of only 63%. Therefore, the authors suggest IMQ treatment of nasal BCCs only if the patient cannot tolerate other types of treatment [68]. After a mean follow-up of 70 months, only 2 relapses were observed among 21 patients with complete response. These 2 relapses were diagnosed with metatypical pathology. Metatypical BCC is a rare subtype of BCC, characterized by both basaloid and squamoid differentiation. The authors suggested that IMQ treatment should be avoided in metatypical carcinoma, due to its aggressive biology. Usually, the prognosis for this type of carcinoma is worse than for the classical BCC, and the recurrence rate is higher [68]. The vast majority of recurrences of the BCC occurred within the first 12-24 months [46]. Moreover, development of SCC on 3 BCC lesions treated with vismodegib, a hedgehog pathway inhibitor, has been reported. The most probable theory is that either the initial lesion was a metatypical BCC or the hedgehog pathway inhibitor may have induced squamous differentiation in some stem cells, located in the deep epidermal layer or near the follicular bulge [68].

There is some evidence in the literature that IMQ can be successfully used in the treatment of some sclerodermiform and infiltrative types of BCC and may induce partial remission of multiple BCCs in patients with Gorlin syndrome or xeroderma pigmentosum [78].

#### 1.1.2. Imiquimod and AK

Oyama et al. showed that AKs which responded to topical treatment with IMQ presented an increase in CD117-positive cells in the dermis. Also, it is important to note that CD117 is present in melanocytes and mast cells. Studies have also shown that the higher the inflammation induced by IMQ, the faster the AKs are eradicated [49, 79].

Therapeutic strategy is chosen based on patient preference and doctor recommendations. When facing a patient with multiple AKs, the treatment of choice is the “field treatment,” using photodynamic therapy, topical chemotherapy, and immunotherapy, this way also treating subclinical AKs [1, 23, 24, 80]. A phase II study showed that topical IMQ 5%, applied 1-3 times/week, significantly reduced the number and dimension of AKs/patient. There were minimal adverse reactions, the therapy being better tolerated than other topical/surgical treatments in use. These findings accompanied by patient education might reduce the morbidity and mortality from SCC, successful treatment of AK making it hard to evolve to aggressive forms of SCC. There is still the need to further study this therapeutic option, to compare it to the gold standard treatment at the moment in order to securely use it [1]. When facing a patient with AK, studies showed that its efficacy ranges from 45.1% to 57.1%, with no significant difference between the number of applications/week (2 vs 3 applications/week) [81–83]. There are some clinical trials that showed comparable efficacy between photodynamic therapy and IMQ cream [73, 84, 85]. A recent study showed that IMQ cream 3.75% was a safe and effective treatment option for AKs, providing complete clearance of AKs in 36% of subjects in phase 3 studies [59]. However, until more information is available, Goh suggests that surgical excision or radiotherapy remains the recommended therapeutic option for such potentially aggressive tumors, because there is a risk of incomplete clearance [86]. Currently, the recommendations are two applications/week for about 16 weeks, but it may vary [11].

#### 1.1.3. Imiquimod and SCC

Ooi et al. showed that the immune response induced by topical IMQ 5% is similar in SCCs and AKs, by increasing the number of CD8^+^ and CD68^+^ cells. *In situ* SCC can be really hard to differentiate from AK, and the fact that the mechanism of healing includes the same paths when treated with topical IMQ 5% means that topical therapy might be a valid alternative to surgical excision [19].

A couple of published case reports and small series have documented IMQ's off-label use in the treatment of in situ SCC, Bowenoid papulosis, extramammary Paget's disease, melanoma *in situ*, cutaneous metastases of melanoma, keratoacanthoma, and others [46].

Huang et al. studied the effects of IMQ therapy on effector T cells infiltrating human SCC, based on the theory that tumor destruction and formation of immunological memory are ultimately T cell-mediated effects. These effector T cells from treated SCCs produced more IFN-*γ*, granzyme, and perforin and less IL-10 and TGF-beta than the cells from untreated tumors. Moreover, the normal skin treated with IMQ presented an activation of resident T cells and a reduced production of IL-10, but no changes on IFN-*γ*, perforin, and granzyme, meaning that these events arise from the recruitment of different populations of T cells. An important aspect was that the blood vessels in human SCC lack E-selectin, evading the skin-homing effector T (Teff) cells and at the same time recruiting Treg cells which can suppress the immune responses. IMQ, the TLR-7 agonist, indirectly addresses both of these mechanisms. This study concluded that the IMQ-treated SCCs were infiltrated by CD8^+^ T cells, which are associated with tumor cell apoptosis and histological signs of tumoral regression [86]. Although there was a shift in the CD4^+^/CD8^+^ cell ratio from 1 : 1 in untreated tumors to 1 : 10 in the IMQ-treated tumors, this was not due to a local proliferation, but most probably from an influx of T cells from the vascular compartment. Another interesting observation is that the treatment of cutaneous Teff cells *in vitro* with IMQ increases the activation and reduces IL-10 production, but it has no effect on IL-17 and IFN-gamma. Moreover, the T cells isolated from the human skin treated for 1 week expressed increased CD69 and decreased CD25 [86, 87].

As mentioned before, untreated SCCs do not express E-selectin and are populated by noncutaneous central memory T cells, 50% of which are FOXP3^+^ Treg cells. IMQ induces vascular E-selectin and recruits tumor-specific CLA^+^ skin-homing T cells. This will lead to a dilution of the Treg cells resident in the tumor and an activation of the tumor-specific CLA^+^ skin-homing T cells within the tumor resulting in a production of IFN-*γ*, perforin, and granzyme and in tumor cell destruction [87].

IMQ induces the local production of IL-6 by nonregulatory Teff cells, therefore making them resistant to suppression. IMQ also reduces Teff production of IL-10 and TGF-beta, thereby reducing tonic inhibitory signals within the tumor. IMQ has an effect on the Treg cells making them reduce their ability to suppress through cytokine production (IL-10, TGF-beta) and contact suppression (CD39, CD73) [87].

Non-Treg cells in untreated SCC are an important source of IL-10, which is also produced by tumor FOXP3^+^ Treg cells. Although some short-term trials have found that IMQ is useful in the prevention of SCC in transplant recipients, the long-term effects of IMQ in these cases is yet unknown [87].

A recent case report presented two cases of SCC treated with once daily application of 5% IMQ cream for 6 weeks. The first patient presented two months later with a subcutaneous nodule, which was histologically diagnosed as recurrent SCC, and after five months following the excision he developed metastatic SCC to a cervical lymph node. The second patient had low-grade chronic lymphocytic leukaemia with SCC in situ of the leg that failed to clear clinically at the end of the IMQ treatment, and after 4 months he re-presented with a focus of invasive SCC within the lesion. In this second case, there was a theoretical potential for failure of immune upregulation with IMQ therapy in immunosuppressed patients. Nonetheless, in the largest study to date, there was a complete clinical and histological response in 14 out of 15 patients with SCC in situ after IMQ topical treatment, once daily for 6 weeks [86].

#### 1.1.4. Imiquimod and Melanoma

It has been reported that IMQ may upregulate gene expression of endogenous angiogenesis inhibitors in melanoma tissue [59]. Off-label, topical IMQ is suggested as an alternative treatment to melanoma surgery and also as an adjunctive therapy after surgery. Topical IMQ has been used recently in the treatment of melanoma *in situ* and also cutaneous melanoma metastases [88, 89]. One case report concluded that 5% IMQ may be used in combination with topical 5-FU in cases of melanoma metastases [90].

Recent studies demonstrated the use of IMQ as an adjunctive therapy for melanoma alongside radiotherapy, by enhancing cell death through autophagy. An overexpression of the autophagy-related genes and also a large number of autophagosomes in B16F10 and B16F1 cell lines were noticed. Apparently, the autophagy was amplified via the ROS-mediated MAPK (mitogen-activated protein kinase) and NF-*κ*B (nuclear factor-kappa B) signaling pathway. Moreover, there was an upregulation of CD8^+^ T cells and a downregulation of Treg cells and myeloid-derived suppressor cells in the tumor lesions. Thus, this study states that IMQ may be used as a radiosensitizer and immune booster alongside radiotherapy for melanoma cases [41, 91].

IMQ alone or in combination with intralesional IL-2 may be a promising immunomodulatory treatment as adjuvant topical treatment for patients with multiple cutaneous melanoma metastases [89].

Some studies suggest that the association between IMQ and BCG (Bacillus Calmette-Guérin) vaccine induces systemic anti-melanoma immunity. The multiple pattern recognition receptor agonists present in BCG and IMQ may prove sufficient to stimulate an immune response against autologous tumor antigens [88]. There is a phase II, single-centre, randomized pilot study which started in 2017, regarding the use of topical IMQ or diphenylcyclopropenone for the management of cutaneous in-transit melanoma metastases [92].

Recent studies have suggested that it can also be used as an alternative treatment for conditions such as malignant melanocytic proliferations and Kaposi's sarcoma [59, 73].

### 1.2. 5-Fluorouracil

**Figure 2:**
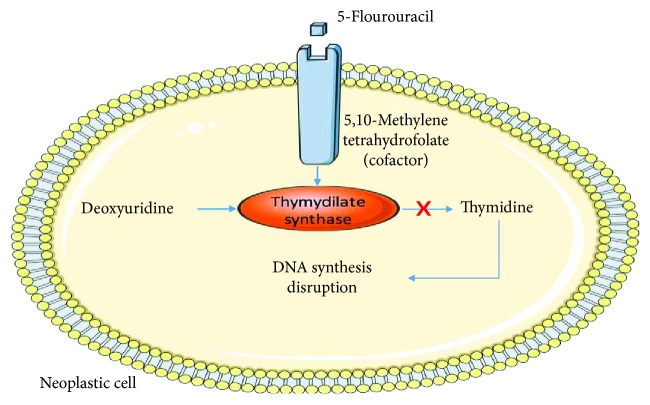
5-Fluorouracil mechanism of action.

5-Fluorouracil (5-FU) belongs to a specific drug class, anti-metabolites. It induces cellular death in cells with high mitotic activity. The main mechanism implies that 5-FU binds to thymidylate synthase through the cofactor 5,10-methylenetetrahydrofolate, causing irreversible inhibition of thymidylate synthase and preventing conversion of deoxyuridine to thymidine. Therefore, DNA synthesis in the neoplastic cells is diminished, leading to a decreased cell proliferation and promoting apoptosis (Figure 2) [93].

#### 1.2.1. 5-Fluorouracil and BCC

Recent data suggest that 5% 5-FU cream may be used in the treatment of superficial BCC, with good cosmetic outcome, no scarring, and only mild erythema [94]. However, this treatment should be limited to patients with small tumors in low-risk locations which cannot undergo first-line therapies. Long-term clinical follow-up is recommended. The recommended regimen is two applications per day, for about 11 weeks with an average of a three-week period of follow-up [95].

#### 1.2.2. 5-Fluorouracil and AK

There is a large number of studies which demonstrate that treatment with topical 5-FU is efficient in AKs. One study showed that 34.8% of the patients treated with 0.5% topical 5-FU and 49% of the ones treated with 5% topical 5-FU reached clinical clearance, while other studies concluded that one application/day of 0.5% for 4 weeks induced complete clearance of 47.5%-57.8% patients [96–99]. Loven and his colleagues showed that both 0.5% and 5% 5-FU have the same rate of complete clearance of 43% of patients [100]. Recent data points out that the severity of AK lesions in patients with organ transplants is significantly reduced after topical use of 5% 5-FU and 5% IMQ, although the treatment is usually longer in these subjects, because skin inflammation, which has an important role in the therapeutic effect, is usually difficult to objectify [101].

After topical use of 5-FU on AK lesions, the expression of keratin 16 was increased; a recent study suggested that proinflammatory cytokines such as IL-1 beta and TNF would be induced after the epidermal injury following 5-FU topical treatment. A two-fold increase of IL-1 beta mRNA was noticed in these cases. Moreover, MMP-1 cleaves the fibrillar type I and II collagens, major structural proteins of the dermis that can be degraded by MMP-3 and MMP-9. Also, MMP-1 mRNA was significantly increased after topical 5-FU treatment, followed by MMP-3 mRNA induction [102].

Creams and solutions are currently available in a range of concentrations, every formula containing different substances that enhance skin penetration. One of the formulas contains salicylic acid, a keratolytic agent, and also a penetration enhancer, dimethyl sulphoxide. Recent studies propose that microsponge formulations are better at depositing more products in the skin, compared to the available formulations [103]. Current treatment regimens suggest one to two applications/day, 2-4 weeks, for the 0.5% fluorouracil cream, in the treatment of AKs [11].

#### 1.2.3. 5-Fluorouracil and SCC

Neugebauer et al. showed that even though in the long term there is no significant difference regarding SCC evolution, 5-FU is more efficient than IMQ in the short term, findings sustained by other studies [104]; therefore, 5-FU might have higher chances of stopping the progression to SCC [24]. The difference of efficiency might be due to differences in their mechanisms of action. IMQ is a synthetic immune modifier, which through TLR-7 activates the innate and acquired immune responses, while 5-FU inhibits cell proliferation and DNA and RNA synthesis, which may have a longer effect than the immune response [24, 81].

Love et al. recommend the use of topical 5-FU, twice daily for 8 weeks, but only for SCC in situ, limited to the trunk, extremities, and neck, smaller than 2 cm, if the patient cannot undergo the first-line treatment. It is not recommended for invasive SCC [95].

### 1.3. Ingenol Mebutate

**Figure 3:**
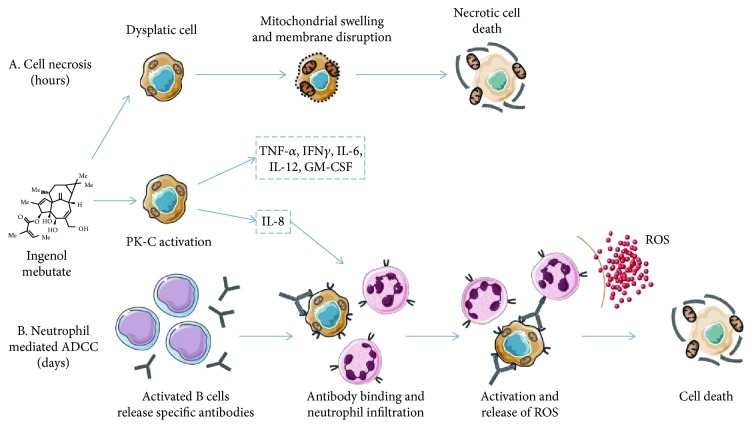
The dual mechanism of action of IM. (a) Rapid necrotic cell death occurring just hours after its application. (b) Neutrophil-mediated antibody-dependent cellular cytotoxicity occurring days after application of the drug. ADCC: antibody-dependent cellular cytotoxicity; GM-CSF: granulocyte-monocyte colony-stimulating factor; IFN: interferon; IL: interleukin; PK-C: protein kinase C; ROS: reactive oxygen species; TNF: tumor necrosis factor.

Ingenol mebutate (IM) is an agent extracted from the sap of *Euphorbia peplus*, a plant which has been used in the past by Romans and Greeks [105], and is recently used in the treatment of various skin diseases such as warts and AK. This molecule was approved for the treatment of AK in 2012, therefore being among the newer topical therapies for skin cancer. It is suggested that there are multiple mechanisms of action, including direct cell death and a complex inflammatory response, mediated partially by PK-C (protein kinase C) activation [11, 106]. Studies have shown that there are two possible ways of inducing cancer clearance. It seems that IM stimulates the production of tumor-specific antibodies and proinflammatory cytokines, therefore inducing cellular cytotoxicity and preventing recurrence [90, 107] (Figure 3).

IM dissolves into the cell membrane and induces a rise in the intracytoplasmic calcium level which then induces mitochondrial destruction [108–110]. After topical application, it produces a neutrophilic infiltration, due to the PK-C activation [111]. The PK-C activation stimulates proinflammatory cytokine production, expression of endothelial adhesion molecules, and tumor-specific antibody formation resulting in a neutrophil-mediated antibody-dependent cellular cytotoxicity [93]. Six hours after the first application of IM, mitochondrial swelling was observed on transmission electron microscopy, and total cell destruction was identified 24 hours after the first application [112]. The inflammatory response induced by this molecule seems to be a T cell-independent effect, with the recruitment of neutrophils which then stimulates the production of ROS [81, 107, 113–115].

The importance of neutrophils in sustaining tumor-free skin is evidenced by a study which showed that in neutrophil-depleted mice, although clearance of the tumor was achieved after 3 days of treatment, the recurrence appeared after 25 days since the treatment with IM [81, 107].

Cozzi et al. showed that topical administration of IM induces the destruction of epidermis, the new epidermis showing significant reduction in keratinocytes expressing p53 mutated gene [116]. It has also been discovered that skin which has not been exposed to UV radiation is less susceptible to develop erythema after topical administration of IM. The mechanism is unknown at the moment, but it is believed that normal skin may not be as permeable to this molecule as sun-damaged skin; also, in normal skin, mast-cell degranulation is lower than in chronic UV-exposed skin [116–118].

#### 1.3.1. Ingenol Mebutate and BCC

IM gel therapy has proved its efficiency without important side effects in the treatment of pigmented and nonpigmented superficial BCC. These results were observed using histology and dermoscopy methods [111]. In a phase IIa trial which evaluated its use in the treatment of superficial BCC, only the highest concentration (0.05%) administered on consecutive days was statistically more efficient than the vehicle [111]. Additional trials are needed because the indications for BCC treatment are currently off-label [11].

#### 1.3.2. Ingenol Mebutate and AK

Another recent study on the pharmacodynamics of IM, and looking at the local changes in both normal skin and in AK lesions on which they applied the drug, suggested that a strong inflammatory response was noted in both instances. There was a heavy T cell infiltration (CD4^+^, in particular) in the papillary dermis as well as neutrophil and ICAM-1 (intercellular adhesion molecule-1) expression on the vascular endothelium of the normal skin. Also, some extravasated erythrocytes were observed in the dermis of some samples of the normal skin but, more importantly, in all of the AK lesions at the end of the treatment. Moreover, the drug modified the expression of numerous genes in both cases and, in particular, in the treated AK lesions, those involved in epidermal development being downregulated. Therefore, they concluded that IM gel 0.05% is capable of inducing epidermal cell death and also immune reactions [119]. The current treatment recommendations are one application of 0.05% or 0.015% gel/day for 2-3 consecutive days [11].

Phase 3 studies showed its efficiency in clearing AK, with sustained clearance over 12 months, using concentrations of 0.015% for face and scalp and 0.05% for trunk and extremities [111]. There is evidence to suggest that IM has higher efficacy than diclofenac 3% and IMQ 5% in the treatment of AK [120].

A case report showed full clinical remission of multiple AKs with good aesthetic outcome in a patient with organ transplant, which used IM on large skin areas. This suggests that IM may be used on large areas, even on 100 cm^2^ of skin, resembling field cancerization treatment by photodynamic therapy without the systemic side effects [121]. There is also evidence that IM treats subclinical lesions present in photodamaged skin and reduces the number of tumors that develop in UV-exposed skin [106]. Treatment efficacy depends on number of consecutive days of application (2 vs 3), region (trunk vs face), and concentration (0.015% vs 0.05%), but the overall sustained clearance at 12 months ranges from 44% to 46.1% [122–124].

#### 1.3.3. Ingenol Mebutate and SCC

Another situation in which IM may be of use is the treatment of multiple SCC in patients with organ transplant, where field cancerization is common, because the immunosuppression promotes keratinocyte tumoral formation and decreases the immunity. Nonetheless, the treatment of field cancerization is very challenging, especially in those with organ transplants [122–124].

Erlendsson et al. have concluded that repeated field-directed treatments with IM delay the development of UV-related SCC in hairless mice [125]. The authors also noticed that increased local skin reactions including erythema, flaking, crusting, vesiculation, swelling, and ulceration are associated to improved clinical outcomes. Currently, it is used off-label in the treatment of SCC [125].

#### 1.3.4. Ingenol Mebutate and Mycosis Fungoides

A 2016 study concluded that topical IM 0.05% may be an effective alternative topical treatment for localized plaques/patches of mycosis fungoides (MF) and folliculotropic MF. It must however be taken into consideration that patients included in this trial were also receiving systemic methotrexate. The authors supposed that the mechanism of action is based on the PMN (polymorphonuclear neutrophil) oxidative burst and keratinocyte cytokine release and, nonetheless, apoptosis. No TCR (T cell receptor) rearrangement was observed in any of the biopsies [126].

Studies have shown that the adherence to IM therapy is higher than with other topical molecules, due to the shorter treatment duration [127–130].

### 1.4. Nonsteroidal Anti-inflammatory Agents and NMSCs

Cyclooxygenase (COX) is an enzyme which limits the production of prostaglandins from arachidonic acid. Topical therapy with nonsteroidal anti-inflammatory agents (NSAIDs) has proven to induce apoptosis, and it seems that there is a very strong link between COX2 activity and the expression of antiapoptotic proteins [131]. COX exists in two forms, COX1 and COX2; the first is constitutively expressed, while the second is expressed after inflammatory stimuli, like ultraviolet light exposure [106, 132, 133]. The overexpression of COX2 has been revealed in numerous neoplasms, including skin cancer. Normal skin has low levels of COX2 and PGE2 (prostaglandin E2), but these levels increase with the severity of the malignancy. Recent studies suggest the importance of COX2 and its products, especially PGE2, in the development of NMSC. Studies show positive results after treatment with NSAIDs for different types of cancer. The main mechanism of action is the inhibition of angiogenesis and the stimulation of apoptosis through COX2 inhibition. Selective inhibition of COX2 in preferred due to the minimal damage to the gastrointestinal tract. In particular, celecoxib, a COX2 inhibitor, has proved its potential therapeutic effect in the prevention of skin neoplasia. Both oral and topical celecoxib have shown chemopreventive effects in animal studies by inhibiting new tumoral formation and delaying tumor latency [106]. There is a strong relation between COX2 and the expression of antiapoptotic proteins of the Bcl-2 family; therefore, the NSAID treatment may induce cellular apoptosis [11].

Diclofenac, a NSAID, reduces the production of prostaglandins by inhibiting the formation of COX2, thereby reducing dysplastic keratinocytes in cancerous lesions [106]. Other mechanisms are the induction of apoptosis by sensitizing neoplastic keratinocytes for ligand induced death, and it is also responsible for the inhibition of angiogenesis in the cancerous cells [93]. Currently, it is approved for the treatment of AK, twice-daily application, for 2-3 months. It can be used including in solid organ transplant recipients, but there are no data regarding its efficacy for BCC or SCC. Two case series have reported clearance of Bowen's disease in a total of 7 patients treated with topical diclofenac for 56 to 90 days. Further studies should be conducted before it can be recommended as treatment for NMSC [106, 134]. Diclofenac also seems to be a valid therapy option for melanoma skin metastases [11, 135].

Currently, the formula containing 3% diclofenac in 2.5% hyaluronic acid has been approved for the treatment of AK in the USA [106], its efficacy ranging from 38% to 47% complete clinical clearance of AKs in different studies [136, 137].

### 1.5. Immunomodulatory Benefits of Drug Associations in Skin Cancer

It has been shown that the efficacy of IMQ can be accentuated by combined therapy with checkpoint inhibitor cytotoxic T lymphocyte antigen (CTLA) 4, of which ipilimumab (a CTLA-4 specific antibody) has shown promising results in metastatic melanoma patients [138, 139]. This antibody seems to be in competition with CD28 during T cell activation [140, 141]. Associated with systemic acitretin, topical IMQ 5% seems to reduce the recurrence of superficial BCC, more than IMQ 5% cream used alone [142]. Rausch et al. showed that IMQ induces a delay in tumor growth and it does not contribute to any memory formation, but by combining it with other immune stimulants like UV-light and CD40 ligands, this inconvenience might be solved [143–145].

5-FU may be applied to the lesion alongside tretinoin cream, which enhances its actions [146].

### 1.6. Novel Therapies and Future Directions

852A (N-[4-(4-amino-2-ethyl-1H-imidazo[4,5c]quinolin-1-yl)butyl]methanesulfonamide, 3 M-001), a small-molecule imidazoquinoline, similar to IMQ, which activates TLR-7 with highly selectivity, is currently being investigated for the treatment of various neoplasms, including inoperable melanoma [42].

Preclinical studies have also demonstrated that IMQ and resiquimod amplify the antitumoral effect of some vaccines by stimulating the innate immune system, but further investigation should be conducted in order to find novel therapies targeting TLR [147].

While some recent data suggest microneedling mediated delivery of diclofenac [148], another important matter is the development of better strategies for the topical delivery of the drug to AKs. Topical therapy is usually used if the tumors are present in the upper layers of the skin and for palliative reasons [59, 103]. There is some data suggesting that iontophoresis may be a good delivery method for IMQ, but the study was only conducted on mice [103].

Further directions should also be oriented towards the bacterial enzyme T4N5 endonuclease, which repairs UVA-damaged DNA. It is a local therapy which was used to treat diseases such as xeroderma pigmentosum, AKs, and BCCs, reducing the lesions [149]. This enzyme is able to minimize the production of cutaneous IL-10 and TNF-alpha and also to restore the interferon-gamma-induced ICAM-1 expression in the skin [150, 151].

## 2. Conclusions

As mentioned above, inflammation not only plays an important role in tumoral growth but also can be used to fight against neoplastic processes.

This analysis of current literature provides an insight into the links between inflammation and cancer.

Since inflammation is known to play a crucial role in the development of skin cancer, this review focuses on topical therapies targeting the inflammation processes occurring in cutaneous carcinogenesis. These therapies usually have minimal adverse reactions, good tolerance, and adherence to the treatment.

Currently, various associations have shown a superior success rate than monotherapy, such as systemic acitretin and topical IMQ or topical 5-FU with tretinoin cream. Another promising combination is IMQ with checkpoint inhibitor cytotoxic T lymphocyte antigen, such as ipilimumab. Novel therapies targeting TLR-7, but with higher selectivity than IMQ, are of great interest.
